# Analysis of Complete Genome Sequence of *Acinetobacter* *baumannii* Strain ATCC 19606 Reveals Novel Mobile Genetic Elements and Novel Prophage

**DOI:** 10.3390/microorganisms8121851

**Published:** 2020-11-24

**Authors:** Mohammad Hamidian, Lucia Blasco, Lauren N. Tillman, Joyce To, María Tomas, Garry S. A. Myers

**Affiliations:** 1 The iThree Institute, University of Technology Sydney, Ultimo 2007, NSW, Australia; Lauren.Tillman@student.uts.edu.au (L.N.T.); joyce.to@uts.edu.au (J.T.); garry.myers@uts.edu.au (G.S.A.M.); 2 Microbiology Department-Research Institute Biomedical A Coruña (INIBIC), Hospital A Coruña (CHUAC), University of A Coruña (UDC), 15495 A Coruña, Spain; lucia.blasco@gmail.com (L.B.); MA.del.Mar.Tomas.Carmona@sergas.es (M.T.)

**Keywords:** *Acinetobacter baumannii* ATCC 19606, complete genome, plasmid, antibiotic resistance, ST52 and Multi-Locus Sequence Typing (MLST)

## Abstract

*Acinetobacter baumannii* isolate ATCC 19606 was recovered in the US prior to 1948. It has been used as a reference and model organism in many studies involving antibiotic resistance and pathogenesis of *A. baumannii*, while, until recently, a complete genome of this strain was not available. Here, we present an analysis of the complete 3.91-Mbp genome sequence, generated via a combination of short-read sequencing (Illumina) and long-read sequencing (MinION), and show it contains two small cryptic plasmids and a novel complete prophage of size 41.2 kb. We also characterised several regions of the ATCC 19606 genome, leading to the identification of a novel cadmium/mercury transposon, which was named Tn*6551*. ATCC 19606 is an antibiotic-sensitive strain, but a comparative analysis of all publicly available ST52 strains predicts a resistance to modern antibiotics by the accumulation of antibiotic-resistance genes via plasmids in recent isolates that belong to this sequence type.

## 1. Introduction

*Acinetobacter baumannii* is a Gram-negative opportunistic pathogen that has emerged in recent decades as a global challenge to healthcare. It causes pneumonia, wound, soft tissue and bloodstream infections and is a major cause of infections in intensive care units (ICUs) [1]. Eradicating *A. baumannii* is problematic mainly because of its natural resistance to extreme environmental conditions and its ability to acquire a range of antibiotic resistance genes [2,3,4]. *A. baumannii* strains have highly plastic genomes, which elevates a need for knowledge of the genomic features at the strain level.

*A. baumannii* ATCC 19606 was recovered in a urine sample prior to 1948 in the US and is one of the earliest isolates available in current collections [5]. It is one of the most antibiotic susceptible strains available to researchers and, hence, has been widely used in studies both as a reference and model strain for studying the emergence and evolution of resistance, pathogenesis and for the discovery of new antibacterial targets [6,7].

Several draft genomes are available for ATCC 19606 (GenBank acc. nos. JMRY01000000 [8], ACQB01000000 and APRG01000000). We deposited the first complete genome for ATCC 19606 in GenBank in October 2019 (GenBank acc. no. CP045110), but in quick succession, two further complete genomes for ATCC 19606 were also made publicly available (GenBank acc. nos. CP046654 and AP022836). One of these genomes was completed using PacBio only (GenBank acc. nos. CP046654) [9], and the latest genome was completed using a combination of Illumina MiniSeq (short-read) and MinION (long-read) sequence data (GenBank acc. nos. AP022836) [10]. Zhu et al. performed a comparative analysis of ATCC 19606 with 98 diverse *A. baumannii* genomes belonging to a variety of sequence types (ST) [9], while the study of Tsubouchi et al. included no analysis of this genome [10].

Here, we report a complete genome for ATCC 19606 generated from a combination of Illumina MiSeq and Oxford Nanopore (MinION) sequence data, as well as an analysis of its genomic features and other ST52 strains. To complement our genome and strengthen the confidence in inferences made from our genomic analysis, we also performed a set of phenotypic assays for antibiotic resistance, mercury resistance, biofilm production and phage lytic ability. Our complete genome for ATCC 19606 includes two cryptic plasmids and a prophage never previously described. Here, we aimed to provide a detailed characterisation of the genetic features and evolutionary relationships of ATCC 19606 and other ST52 strains to further understand the genomic features and mechanisms for the development of antibiotic resistance.

## 2. Materials and Methods

### 2.1. Antibiotic Resistance Profile and Resistance to Mercury

Antibiotic resistance profile of *A. baumannii* ATCC 19606, which was obtained from the ATCC culture collection and kindly supplied by Prof. Ruth Hall of the University of Sydney, Australia, against 22 antibiotics was determined using the standard disc diffusion method, as previously described [11]. Antibiotic discs tested were: ampicillin (25 µg), ampicillin/sulbactam (10/10 µg), cefotaxime (30 µg), ceftazidime (30 µg), imipenem (10 µg), meropenem (10 µg), piperacillin/tazobactam (100-10 µg), timentin (ticarcillin/clavulanic acid) (75/10 µg), streptomycin (25 µg), spectinomycin (25 µg), sulfamethoxazole (100 µg), trimethoprim (5 µg), kanamycin (30 µg), neomycin (30 µg), gentamicin (10 µg), amikacin (30 µg), tobramycin (10 µg), netilmicin (30 µg), rifampicin (30 µg), tetracycline (30 µg), ciprofloxacin (5 µg) and nalidixic acid (30 µg). Resistance and susceptibility were interpreted according to the Clinical and Laboratory Standards Institute (CLSI) guidelines for *Acinetobacter* spp. [12] and calibrated dichotomous sensitivity disc diffusion assay (CDS) (http://cdstest.net/) when a CLSI breakpoint for *Acinetobacter* spp. was not available (e.g., for netilmicin, streptomycin, spectinomycin, sulfamethoxazole, nalidixic acid and rifampin). Antibiotic resistance profile was determined using two individual colonies of ATCC 19606. All antibiotics were purchased from OXOID, UK.

To examine whether ATCC 19606 is resistant to mercury, 10 fresh colonies were patched onto L-agar supplemented with 20-μg/mL HgCl2 followed by overnight incubation at 37 °C and visual inspection for the presence and absence of growth, as previously described [13].

### 2.2. Static Biofilm Assay and Quantification of Biofilm Production Using Fluorescence Microscopy

Biofilm formation was measured using the standard crystal violet assay in 96-well plates, as described previously [14]. Briefly, ATCC 19606 and controls were grown overnight at 37 °C in Cation-adjusted Muller-Hinton broth (CaMHB; Sigma-Aldrich, St. Louis, MO, USA). All cultures were then normalised to OD600 of 0.05 (~1 in 100 dilution) and then 100 μL of cells transferred to each well of a 96-well plate. Growth and media-only controls were also included. The 96-well plate was covered with Aeroseal (Thermo Fisher^TM^, Waltham, MA, USA) and incubated for 24 h and a second plate for 48 h at 37 °C. After the incubation, plates were washed with PBS buffer (Phosphate-buffered Saline) using an automatic plate washer (BIO-TEK ELx405TM, Winooski, VT, USA), followed by staining with 150 μL of 0.2% CV (Crystal Violet) per well and incubating at room temperature for 1 h on an automatic rocker. The washing step was repeated, followed by adding 170 μL of 33% acetic acid to each well and incubating at room temperature on a rocker for 30 min. Absorbance at 600 nm was measured using a plate reader (Tecan M200, Männedorf, Switzerland).

Fluorescence microscopy was used to visualise biofilm microcolonies and to measure their properties. Briefly, 1 mL of normalised cell culture (as prepared for CV assay) was grown for 24 or 48 h at 37 °C in CaMHB in WPI^TM^ dishes. After the incubation, dishes were washed gently with saline and stained with 1 mL of 2-uM Syto-9 for 1 h, then washed again and fixed with 1 mL of freshly prepared 4% PFA for 1 h prior to imaging. DeltaVision Elite microscope (Applied Precision) was used to acquire Z-stack wide-field fluorescence images; 5 randomly selected fields of view (FOV) were captured in the FITC channel at 20× magnification. All images were analysed using IMARIS softwarev.9.5.1 with the Biofilm Analysis tool (https://imaris.oxinst.com).

All biofilm assays were performed with technical triplicates and included three additional strains, AB0057 (member of global clone 1), ACICU (member of global clone 2) and ATCC 17978 (widely used reference strain). Results were analysed and plotted using the Prism GraphPad software v8.2.0. 2.3. whole-genome sequencing and assembly.

### 2.3. Genome Sequencing

Whole-cell genomic DNA of ATCC 19606 was isolated using the DNeasy^TM^ UltraClean Microbial Kit (Qiagen^TM^, Germantown, MD, USA) from cells grown overnight at 37 °C in LB inoculated from a single colony. Library preparation and barcoding for Illumina MiSeq and MinION (Oxford Nanopore Technologies ^®^, Oxford, UK) sequencing was performed by the UTS Core Sequencing Facility at the ithree Institute, as described previously [15,16]. Illumina sequencing generated 1,024,087 paired-end short reads with 50-fold coverage and an average length of 250 bp; MinION generated a total of 10,687 reads with an N50 of 18.2 kbp and 30-fold coverage. FastQC (v.0.11.9) (https://bioinformatics.babraham.ac.uk/projects/fastqc/) and Filtlong (v.0.2.0) (https://github.com/rrwick/Filtlong) were used to check the quality of Illumina and MinION reads, respectively. Filtlong filtered long reads by quality and length. The high=quality Illumina and MinION reads were assembled de novo using a hybrid assembly approach with the Unicycler program (v0.4.7) [17]. Protein coding, rRNA and tRNA gene sequences were annotated using Prokka [18], and the resistance and polysaccharide loci (outlined below) were annotated manually.

### 2.4. Phylogenetics and Sequence Analysis

Phylogenetic relationships of all available ST52 strains were examined by generating a core genome alignment. Briefly, Illumina sequence reads for all isolates were mapped to ATCC 19606, which was also used as a reference using snippy (available at https://github.com/tseemann/snippy) to generate a whole-genome alignment. Snippy mapped all reads to the reference genome using bwa v0.7.12 and minimap2 v 2.0 using default parameters. High-quality variant sites were called using SAMtools v1.3.1.24 with standard-quality filtering, as described previously (10). Single-nucleotide differences (SNDs) in recombinant regions were identified and removed using Gubbins v2.1.025 (33) with default parameters, including the default taxa filtering percentage of 25%. A maximum likelihood phylogenetic tree was inferred from the resulting recombination-filtered alignment using RAxML (v.8) with the GAMMA model. The tree was visualised and annotated using the R package ggtree v1.12.027. Recombination blocks were plotted against the phylogenetic tree in R v.3.5.2. using the ggtree v. 1.16.6 and ggplot2 v.3.2.1 packages and PlotTree available at https://github.com/katholt/plotTree. Bootstrap values were calculated using ten independent runs of RAxML with 1000 bootstraps, which each gave near-identical results.

A range of bioinformatic tools were used for the sequence analysis. A local database of the genome sequence of the strains studied here was created, and sequence analysis was done locally using the standalone BLAST program available at ftp://ftp.ncbi.nlm.nih.gov/blast/executables/blast+/LATEST/. The Artemis Comparison Tool (ACT) 16.0.0 [19] was used to visualise comparisons of large regions performed by Standalone BLAST. SnapGene Viewer v 4.2.4 was used to visualise, manipulate and export the sequence data.

Protein coding and gene features studied here were annotated manually using a combination of BLASTP (http://blast.ncbi.nlm.nih.gov/Blast.cgi), Pfam (http://pfam.xfam.org/) and UniProt (https://www.uniprot.org) searches, as described elsewhere.

The IS-Finder (https://www-is.biotoul.fr/) database was used to identify and analyse insertion sequences (IS).

The PHASTER (http://phaster.ca) database was used to look for integrated phage genomes. The phage genome sequences were further analysed and annotated using the RAST server (Rapid Annotation using Subsystem Technology; https://rast.nmpdr.org), BLAST (https://blast.ncbi.nlm.nih.gov/Blast.cgi) and the HHPRED bioinformatics toolkit (https://toolkit.tuebingen.mpg.de/tools/hhpred). Figures were drawn to scale using SnapGene Viewer^®^ v 4.2.4 and reconstructed using the Inkscape v.1.0 program.

### 2.5. Data Availability

The complete genome sequence was deposited in DDBJ/ENA/GenBank under the accession no. CP045110 (chromosome), CP045108 (p1ATCC 19606) and CP045109 (p2ATCC 19606). Illumina and MinION sequence reads were deposited in the sequence read archive database under the accession numbers SRR10248709 and SRR10248708, respectively.

### 2.6. Phage Induction and Isolation

In order to induce the expression of the prophage and its release from the bacteria, an overnight culture of *A. baumannii* strain ATCC 19606 was diluted 1:100 in LB broth and incubated at 37 °C and 180 rpm. When the culture reached 0.5 OD (600 nm), mitomycin (10µg/mL) was added to the culture and incubated until the culture was cleared by lysis. After 30 min of incubation at room temperature in the presence of chloroform (1%), the culture was centrifuged at 3000× *g* for 15 min, and the supernatant with the isolated phage was recovered and filtered through a 0.45-um filter.

### 2.7. Phage Concentration and Preparation for Transmission Electron Microscopy (TEM)

To concentrate the phage previously isolated, the lysate was incubated with NaCl to a final concentration of 0.5 M and left on ice for 1 h. The suspension was centrifuged at 3400× *g* for 40 min at 4 °C, and the supernatants were transferred to sterile tubes. PEG 6000 (10% *w/v*) (polyethylene glycol) was added, dissolved and incubated overnight at 4 °C. Bacteriophages were then precipitated at 3400 *g* for 40 min at 4 °C and resuspended in SM buffer (0.1-M NaCl, 1-mM MgSO4 and 0.2-M Tris-HCl, pH 7.5).

For the visualisation of the phage by TEM, the samples of the phage in SM buffer were negatively stained with 1% aqueous uranyl acetate in grids and then examined on a JEOL JEM-1011 electron microscope.

### 2.8. Phage Host Range

The host range of the phage was established by applying a previously established spot test [20] to a set of diverse 19 clinical strains of *A. baumannii*, of different STs, isolated from Spanish hospitals during the GEIH-REIPI Spanish Multicentre *A. baumannii* Study II 2000–2010, GenBank Umbrella project PRJNA422585. Briefly, an overnight culture of the *A. baumannii* strain host was diluted 1:100 and incubated at 37 °C and 180 rpm until it reached an optical density of 0.5 (OD600nm). TA soft medium (agar 0.4%) supplemented with 10-mM CaCl_2_ was previously prepared and maintained at 50 °C. Four millilitres of the TA soft medium was mixed with 200 µl of the *A. baumannii* culture, and the mix was poured on the top of a TA plate (agar 1.5%). When the soft medium was solidified, a spot of 10 µl of the phage suspension was dropped on the plate, and when adsorbed, it was incubated at 37 °C. After 24 h, the plates were checked to detect the presence of a halo, which indicated lysis.

## 3. Result

### 3.1. Antibiotic Resistance Profile

The resistance profile for ATCC 19606 to individual antibiotics has been tested in previous studies. However, to draw a complete picture of ATCC 19606′s resistance profile and to phenotypically confirm the genome reported here, ATCC 19606 was tested against 22 antibiotics representing all clinically important antibiotic classes. Overall, ATCC 19606 is susceptible to a wide range of antibiotics, including nalidixic acid, ciprofloxacin and many antibiotics in the ß-lactam and aminoglycoside families (Table 1). It was found to be resistant to ampicillin, streptomycin spectinomycin, chloramphenicol and sulfamethoxazole (Table 1).

### 3.2. Complete Genome Sequence of ATCC 19606, Antibiotic Resistance Gene and Its Genomic Context

**Figure 1:**
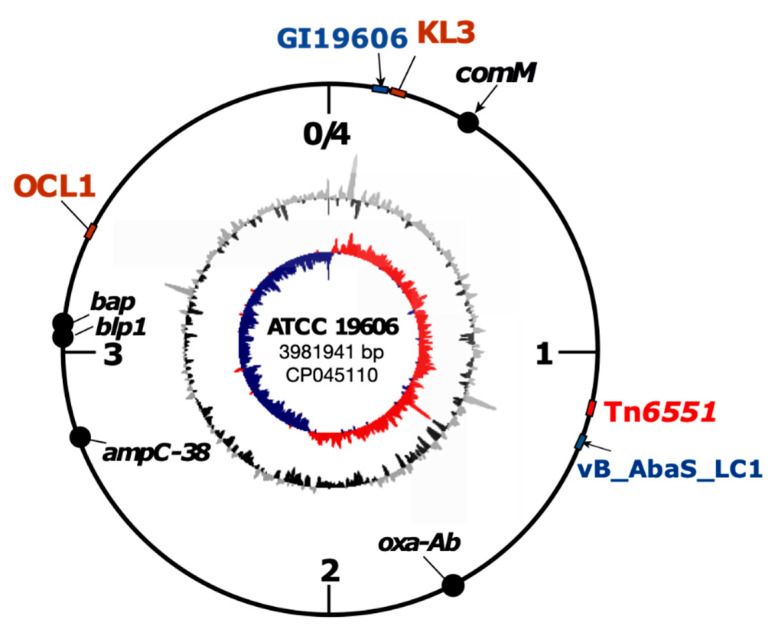
Schematic representation of the complete genome of *Acinetobacter baumannii* ATCC 19606. Important genes, genomic islands, transposons and surface polysaccharide loci (OC and K) are marked on the outermost ring representing the chromosome. The inner most ring represents the GC skew, and the middle ring represents the GC content of the ATCC 19606 chromosome.

To determine the complete genome sequence of ATCC 19606, we used a combination of Illumina and MinION sequence data. We used the long reads to generate the genome scaffold and >50 rounds of polishing using the Illumina reads, given the accuracy of Illumina reads, to minimise the sequencing errors in the final assembly. The genome sequence of ATCC 19606 was completed and submitted to GenBank in October 2019 (https://www.ncbi.nlm.nih.gov/nuccore/CP045110). The final assembly consists of 3,981,968 bases, including the chromosome (3,981,941 bp; Figure 1) and two plasmids, p1ATCC19606 (7655 bp) and p2ATCC19606 (pMAC) (9540 bp), with a copy number of 11x and 13x for each plasmid, respectively. The chromosome of ATCC 19606 has an average GC content of 39.15% (highest 51.66% and lowest 22.5%) and encodes a total of 3693 putative proteins, 74 tRNAs and 6 rRNA regions.

Recently, the genome of ATCC 19606 was also completed by two other groups: (i) using the PacBio technology (Table 2) [9] and (ii) a combination of Illumina MiniSeq and MinION [10]. To examine the differences in the genomes generated by the three groups, we compared all three genomes. The chromosome sequence of our assembly is 1093-bp longer than that in CP046654 due to the absence of a copy of the insertion sequence ISAba11, which was found at positions 998797–999897 of our assembly. The two chromosome assemblies also differ by 50 single-nucleotide differences (SNDs) and small insertions/deletions of mainly one–five bases (50 bp in total) spread across the chromosome. The chromosome of ATCC 19606 deposited in GenBank acc. no. AP022836 differs from our assembly again by the absence of the additional ISAba11 found in our assembly, 270 SNDs and a substantial number of small insertions/deletions (1660 bp in total) of mainly up to 10 bases in the chromosome. Notably, there are far less differences between our assembly and that completed using PacBio by Zhu et al. [9] than the genome reported by Tsubouchi et al. [10]. The additional ISAba11 copy in our assembly is presumably due to IS movement, given that there is already another ISAba11 in all ATCC 19606 chromosomes, while SNDs and short insertions/deletions could be due to either sequencing/assembly errors, real mutations or, likely, a combination of both.

The complete genome of ATCC 19606 includes a single acquired antibiotic resistance gene, the *sul2* sulfonamide resistance gene, which confers resistance to sulfamethoxazole and accounts for the sulfamethoxazole resistance phenotype observed in this strain [21]. We previously predicted, using the publicly available draft genome sequences of ATCC 19606, a chromosomal genomic island in ATCC 19606 containing the *sul2* gene in the GI*sul2* genomic island [21]. This GI will be referred to as GI19606 hereafter. The complete ATCC 19606 genome reported here confirms the structure of GI19606 as a 36,157-bp genomic island located at bases 80477–116633 of the ATCC 19606 chromosome (GenBank acc. no. CP045110) spanning locus_ids FQU82_00080 to FQU82_00125 (Figure 1). The other two ATCC 19606 complete genomes also contain GI19606 [9,10].

ATCC 19606 is susceptible to third-generation cephalosporins and carbapenems, and consistent with this, no ISAba1 copy was found upstream of the intrinsic *ampC* and *oxa-Ab* genes. However, in our complete genome assembly, the ATCC 19606 chromosome contains a copy of the intrinsic *ant(3”)-IIa* aminoglycoside resistance gene (located at bases 225025–225813; locus id FQU82_00218), accounting for the streptomycin and spectinomycin resistance phenotypes observed (Table 1). This intrinsic *ant(3”)-IIa* aminoglycoside resistance gene is also present in the other two complete genomes.

### 3.3. Tn6551, a Novel Mercury/Cadmium Transposon Found in ATCC 19606

**Figure 2:**
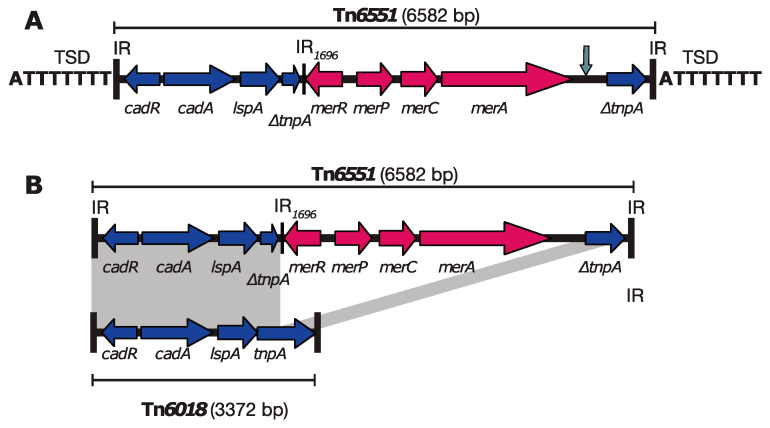
Genetic structure of Tn*6551* (**A**) and its comparison to Tn*6018* (**B**). Horizontal arrows indicate the extent and orientation of genes and open reading frames with their names shown below. Inverted repeats (IR) are indicated using black vertical lines. Target site duplications generated by Tn*6551* are shown flanking this element in panel A and marked target site duplications (TSD). The vertical grey arrow indicates the position of the 96-bp deletion in Tn*6551-v1*.

Given that genes conferring resistance to antibiotics and resistance to heavy metals can occur together on the same genetic element, we searched the complete genome of ATCC 19606 for genetic elements associated with heavy metal resistance. A set of *mer* resistance genes predicted to confer resistance to mercury were found on an approx. 6.5-kbp region that was flanked by a set of cadmium/zinc resistance genes and a partial *tnpA* gene, which were identical to those in Tn*6018* [3]. Tn*6018* is a 3372-bp cadmium/zinc transposon that is often found embedded in the AbaR-type resistance islands in members of global clone 1 [3]. Tn*6018* is flanked by 24-bp inverted repeats (IR) and generates 8-bp target site duplication upon insertion [3]. Further analysis of this region showed that this mercury/cadmium/zinc region in ATCC 19606 includes properties of a class II transposon and is related to Tn*6018*. Hence, this transposon was named Tn*6551* (Figure 2). Tn*6551* is a 6582-bp novel transposon bounded by 24-bp IRs and flanked by the 5′-ATTTTTTT-3′ 8-bp target site duplications (TSD). Tn*6551* is located at bases 1237446–1244027 of the ATCC 19606 chromosome (GenBank acc. no. CP045110) between FQU82_01194 (encoding a hypothetical protein) and the *fic* gene (FQU82_01201, encoding a putative adenosine monophosphate-protein transferase). In Tn*6551*, the 5′-end of the *tnpA* gene is interrupted by the *merACPR* genes (Figure 2). However, the other end of this *mer* module appears to be deleted, as the IR*_tnp_* of Tn*1696* could not be detected. Analysis of the *mer* region indicated that it is a hybrid Tn*501*/*1696*/*5053 mer* module (GenBank acc. no. Z00027: Tn*501*, Y09025: Tn*1696* and L40585: Tn*5053*) with an IR*_1696_* located at the 3′-end of *merR*.

ATCC 19606 was tested for mercury resistance; however, none of the colonies patched onto L-agar supplemented with 20-μg/mL HgCl2 generated a visible colony, suggesting that this mercury operon is likely to be nonfunctional.

To track Tn*6551*, the GenBank nonredundant database was searched using the sequence of Tn*6551* as a query. Tn*6551* and/or variants of it were found in related strains, unrelated strains, different *Acinetobacter* species and a different bacterial genus. A copy of Tn*6551* was found precisely in the same chromosomal location in an ST52 clinical strain recovered in 2015 in the US (strain ab736; Table 3). This suggests a link to ATCC 19606, given that both strains belong to the same sequence type (see a comparative analysis of ST52 strains below). An almost identical copy of Tn*6551* (with 99.9% DNA identity) was identified also in two other completely unrelated *Acinetobacter* strains, AB031 and BEC1-S18-ESBL-01 (Table 3). Notably, the latter belongs to a different species, *A. pittii*, suggesting the transfer of Tn*6551* across species. In addition, a variant of Tn*6551*, which we named Tn*6551-v1*, was found in several strains belonging to different species of the *Acinetobacter* genus, indicating that both Tn*6551* and Tn*6551-v1* are widely spread (Table 3). Compared to Tn*6551*, the *-v1* variant is 96-bp longer and was likely generated due to a separate deletion event truncating the hybrid *mer501*/*1696*/*5053* module, leaving an additional 96 bp of the *mer* module behind (vertical grey arrow in Figure 2A). Alternatively, it is possible that Tn*6551* is a derivative of Tn*6551-v1* that was generated as a result of a second deletion event; however, this could not be verified, as no intermediate structure is currently available in GenBank. Unexpectedly, a copy of Tn*6551-v1* was also found in the chromosome of a Neisseria brasiliensis strain, N.177.16, recovered in 2016 in Brazil (Table 3). N.177.16 belongs to a completely different bacterial genus, suggesting its transposition across two different bacterial genera.

We previously showed that DNA fragments containing an ISAba1-activated *ampC* gene or an entire genomic island could be acquired from an exogenous source via homologous recombination [22,23]. To examine whether homologous recombination is the mechanism for the Tn*6551* and Tn*6551-v1* exchange events, we analysed the chromosomal sequences flanking Tn*6551* and the *-v1* variant. Analysis of the flanking sequences of Tn*6551* showed that a 7044 bp on the left and a 3934 bp on the right of Tn*6551* in ATCC 19606 were 100% and 99.99% (1-bp difference), respectively, identical to the corresponding sequences in AB301 (GenBank acc. no. CP009256). This suggests a possible homologous recombination exchange event of a 17,560-bp DNA fragment (including Tn*6551*) between ATCC 19606 and AB301 (Table 3). AB301 belongs to ST638, which is a very rare sequence type with only one complete genome available in GenBank. No other exchange event was detected, indicating that the acquisition of Tn*6551* and Tn*6551-v1* by different strains of *Acinetobacter* species occurred through multiple transposition events. In all but *A. nosocomialis* AC1530 and *Neisseria brasiliensis* N.177.16, Tn*6551* and Tn*6551-v1* are present in precisely the same chromosomal location, suggesting a preference for this particular chromosomal site. Nonetheless, the presence of Tn*6551-v1* copies in completely different chromosomal locations in *A. nosocomialis* AC1530 and *Neisseria brasiliensis* N.177.16 indicates that these transposons could also target alternative genomic spots.

### 3.4. ATCC 19606 Carries Two Cryptic Plasmids

**Figure 3:**
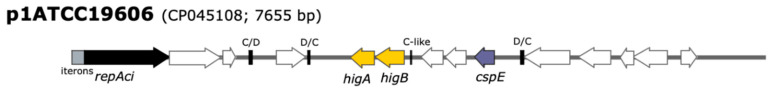
Genetic structure of p1ATCC 19606. Horizontal arrows indicate the extent and orientation of genes and open reading frames with their names shown below. White arrows indicate open readings with unknown functions. Small black vertical lines indicate p*dif* recombination sites, and the filled grey box upstream of the *repAci* gene indicates the iteron region.

Our complete ATCC 19606 genome carries two cryptic plasmids, which are named p1ATCC19606 and p2ATCC19606 (pMAC). In addition to our assembly, the entire sequence of p1ATCC19606 is present in all available draft genomes of ATCC 19606 (GenBank acc. no. JMRY01000000, ACQB01000000 and APRG01000000) and in the complete genome of the Tsubouchi et al. group [10] (Table 2), but it is absent from the genome reported by Zhu et al. [9], likely due to sequencing/assembling error. p1ATCC19606 is a novel 7655-bp cryptic plasmid that encodes 14 open reading frames, one of which is a novel RepAci putative replication initiation protein (Figure 3). Its closest known match is RepAci2 (encoded by pABVA01; GenBank acc. no. FM210331) with 95% aa identity. p1ATCC19606 also encodes the *higAB* toxin-antitoxin system, a prevalent toxin-antitoxin type in small plasmids of *A. baumannii*.

Recently, a number of *A. baumannii* plasmids have been shown to include p*dif* sites, consisting of inversely oriented binding sites for the XerC and XerD recombinases separated by 6 bp [13,24,25,26]. Here, our manual inspection of p1ATCC19606 identified three p*dif* sites (XerC/D at bases 1971–1998, XerD/C at bases 2613–2640 and XerD/C at bases 4973–5000), as well as an 11 bp that resembles a XerC-binding site at bases 3758–3768 of p1ATCC19606 (GenBank acc. no. CP045108; Figure 3). The p*dif* module, carrying *higAB* and flanked by D/C and D/C sites, is in a wide range of small plasmids (data not shown). The sequence of this plasmid in the genome completed by the Tsubouchi et al. group [10] is 24-bp shorter due to the absence of the C/D site of the module carrying a single orf. This might represent a sequencing/assembling error, as careful inspection of the flanking sequence did not reveal any evidence to explain the deletion. However, this could not be verified, as ATCC 19606^T^ was not available.

p2ATCC19606, also known as pMAC, is a well-studied 9540-bp cryptic plasmid first reported in 2006 [27]. The sequence of p2ATCC19606 (pMAC) in our assembly and that sequenced by Zhu et al. [9] are identical to the original sequence of pMAC (GenBank acc. no. AY541809.1) (Table 2). However, the sequence of this plasmid is 132-bp shorter in the sequence reported by Tsubouchi et al. [10], again likely due to sequencing errors.

Our manual inspection of p2ATCC19606 (pMAC) identified five p*dif* sites, including XerC/D at bases 1868–1895, XerD/C at bases 2510–2537, XerD/C at bases 5226–5253, XerC/D at bases 6264–6291 and XerD/C at bases 6809–6836 of GenBank acc. no. CP045109 (picture not shown), which indicates that it consists of five p*dif* modules. This was not noted before.

### 3.5. ATCC 19606 Carries a Novel Prophage, vB_AbaS_LC1

Our analysis of the genomic sequence of ATCC 19606 established the presence of a 41,282-bp complete prophage. This prophage was located at the genomic position 1336408–1377688 bp (GenBank acc. no. CP045110; Figure 1) and had a GC content of 40.4%. This prophage region encodes 59 open reading frames (Table 4), of which 41% have unknown functions; 10% have putative replication, recombination and synthesis functions; 17% are structural proteins; 5% involved in defence against the host; 6% involved in the lytic-lysogenic cycle and 5% have putative functions related to the entry and exit of the virus from the cell, including a lysozyme and a sialidase. These have potential as enzymes for phage therapy.

To find out if there were any homologues of the prophage that we identified, we first performed a homology analysis by BLAST of the prophage sequence. This showed the presence of an identical genomic region in the chromosome of *A. baumannii* strain Ab736 (GenBank acc. no. CP015121). Ab736 belongs to the sequence type ST52 and is, therefore, related to ATCC 19606. However, this genomic region is not annotated and/or reported as a prophage. Secondly, we performed a bacteriophage homology analysis by comparing the genomic sequence of the phage with the viral genomes deposited in the GenBank nonredundant database. No homology was obtained for any known phage, suggesting that the prophage we found in ATCC 19606 is a novel phage. We named this phage vB_AbaS_LC1.

**Figure 4:**
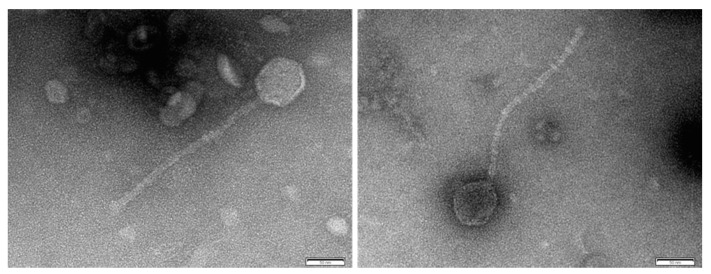
TEM image of the phage vB_AbaS_LC1. Samples of the phage in SM buffer were negatively stained with 1% aqueous uranyl acetate in grids and, then, were examined on a JEOL JEM-1011 electron microscope. Scale bar indicates 50 nm (nanometre).

To determine whether our designation of the genomic region as a novel phage had functional relevance, we isolated vB_AbaS_LC1 after inducing its exit from the bacterial cell with mitomycin. Once vB_AbaS_LC1 was isolated and concentrated, we visualised it by TEM. TEM showed that the phage has a morphotype characteristic of a tailed phage from the Siphoviridae family, with a noncontractile tail of 250 µm and an icosahedral capside of 60 µm (Figure 4). Spot test results showed that vB_AbaS_LC1 has a narrow host range, as it was able to infect only two of the 19 *A. baumannii* clinical strains assayed (Table S1). Each of the infected strains belonged to different STs, namely ST255 and ST265. Only one of the 19 strains assayed belonged to the same ST as ATCC 19606 (Ab22_GEIH2010; ST52), which was not susceptible to the virus.

### 3.6. Biofilm Formation

Biofilm formation is an important property that allows *A. baumannii* to survive on surfaces for a long period of time. To date, multiple genes have been shown to be involved in biofilm formation in *A. baumannii*, including *ompA* (encoding an outer membrane protein), the *csuABCDE* operon (encoding a type I pili) and the *bap* and *blp1* genes, which encode large biofilm-associated proteins and often vary in sizes [4,28]. Differences in the sizes of the proteins encoded by the *bap* and *blp1* genes—differences that impact the presence/absence of domains present in the Bap and Blp1 proteins—have been used to explain some of the variations observed in the biofilm formations of *A. baumannii* strains. Knockouts that lack either of the *bap* and *blp1* genes produce significantly lower amounts of biofilm [4]. The complete genome of ATCC 19606 we report here encodes a 9831-bp *blp1* gene (locus_id FQU82_03028) and an 18,543-bp bap gene (locus_id FQU82_03059). Both differ in size, mainly due to a different number of repeat units, compared to those encoded by AB0057 (GC1), ACICU (GC2) and ATCC 17978, which is another commonly used *A. baumannii* reference strain.

**Figure 5:**
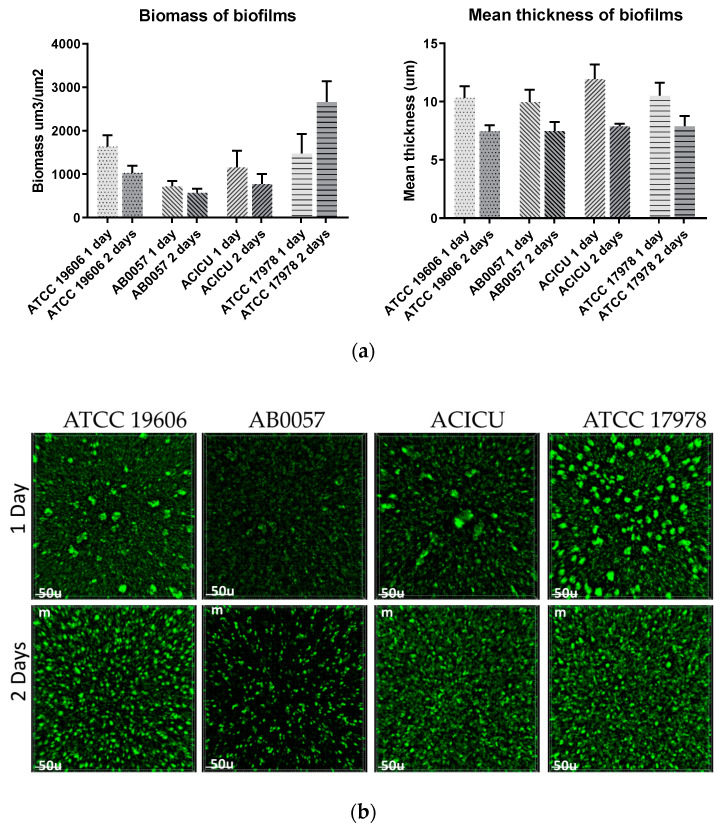
Analysis of the biofilms formed by ATCC 19606, ATCC 17078, AB5007 and ACICU using fluorescence microscopy. (**a**) shows the quantification of biofilm biomass and thickness of ATCC 19606 and controls after 24 and 48 h. (**b**) Fluorescence microscopy images of biofilms stained with Syto-9 and taken using the 20× lens after 24 and 48 h (top).

To complement the complete genome sequence of ATCC 19606 reported here, we quantified the biofilm formation of ATCC 19606 by crystal violet assay and by measuring biomass and thickness using fluorescence microscopy. A member of global clone 1 (AB0057), clone 2 (ACICU) and the widely used reference strain ATCC 17978 were also included in biofilm studies as controls. Using the standard crystal violet assay, and under the conditions examined here, visual inspection showed both ATCC 19606 and ATCC 17978 formed a little biomass at the culture-air interface compared to AB5007 and ACICU, which showed very strong staining around the well. These observations are reflected in the crystal violet measurements of OD600 0.78 and 0.36 versus 3.9 and 3.5, respectively. Using fluorescence microscopy, which captures cells fully submerged in the culture and directly attached to the surface, biofilm macro-colonies could be visualised in all strains after 24 h. ATCC 19606 generated a biofilm biomass of approx. 1600 µm^3^/µm^2^, which was similar to that generated by ATCC 17978 and higher than those generated by AB0057 and ACICU (Figure 5). Interestingly, after 48 h, ATCC 17978 showed the highest increase in biofilm biomass, while the biomass decreased in ATCC 19606 and other controls (Figure 5). Biofilm thickness was measured to be approx. 10 µm for all strains after 24 h, and all biofilms decreased by approx. 20% in the 48-h experiment (Figure 5). Differences in biofilm formations observed in ATCC 19606 compared to other controls maybe explained by there being differences in the size of the proteins encoded by the *blp1* and *bap* genes between the strains tested.

### 3.7. Surface Polysaccharide Loci Types

Surface polysaccharides, including capsular polysaccharides (CPS, K or capsule), play a pathogenic role for *A. baumannii*. These loci are particularly important for the production of surface polysaccharides that function as virulence determinants for *A. baumannii*: the K locus that contains genes directing the synthesis of the surface polysaccharide capsular polysaccharide and the OC locus that contains genes involved in the synthesis of the outer core component of the lipooligosaccharide [29]. Previously, draft genomes of ATCC 19606 were used to predict the presence of the surface polysaccharide loci, including the KL3 capsule biosynthesis and OCL1 outer core [30]. Here, our assembly confirms the KL3 and OCL1 assignments.

### 3.8. Comparative Analysis of ST52 Strains

**Figure 6:**
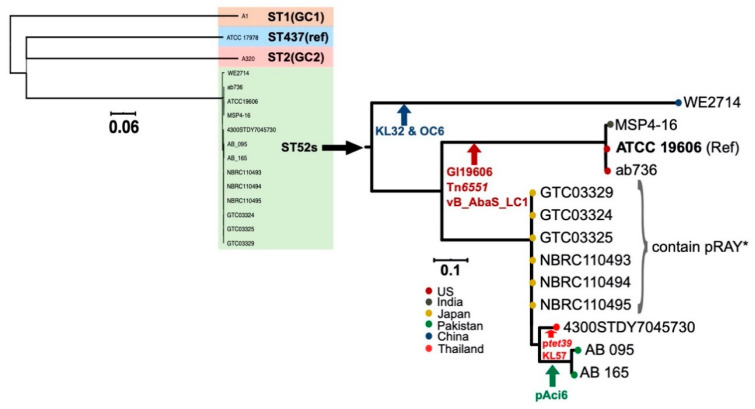
Phylogenetic relationship of ST52 strains to the references GC1, GC2 and ATCC 17978 (**left**) and within the sequence type 52 (**right**). Node colours indicate the country of isolation, and vertical arrows indicate branch-specific acquisition events. All Japanese strains indicated by a curly bracket contain pRAY*. Scale bars are shown.

ATCC 19606 belongs to ST52. To examine the evolution of strains belonging to ST52, GenBank nonredundant and WGS (Whole Genome Shotgun) databases were explored and, then, phylogenetic analysis performed using the genomes found. In addition to ATCC 19606, 12 genomes were found as belonging to ST52 (Table 5). The additional genomes, all recovered after 2008, represent isolates from diverse geographical regions and diverse sources, including soil, clinical samples and hospital environments (Table 5). A phylogenetic analysis using the whole-genome alignment of ST52 strains showed, as expected, that they all cluster into a clade distinct from other major clonal groups, such as ST1 and ST2 (Figure 6).

However, a further analysis revealed a high degree of diversity within this tight phylogenetic group (Figure 6)—in particular, regarding their horizontally acquired resistance determinants. Within the ST52 clade, two distinct subclades were found. The subclade that we hereafter refer to as subclade 1 (SC1) contains a single Chinese strain (WE2714) in a deep branch and distant from the other strains. Subclade 2 (SC2) has two branches. In one branch, ATCC 19606, MSP4–16 and ab736 clustered tightly, indicating their close relationship, despite differences in their sources of isolation, country and isolation date (Table 5). The other branch of SC2 consists of a set of Japanese genomes, along with three additional genomes, one from Thailand and two from Pakistan. These three additional genomes cluster into a separate branch (Table 5 and Figure 6). Interestingly, the Japanese strains differed from each other by <30 single-nucleotide variants across their entire genomes, suggesting that they are likely to be outbreak strains given their isolation source and time (Table 5).

We screened the ST52 strains for additional genetic features that could differentiate/group them. Interestingly, all strains in the ATCC 19606 branch, regardless of their country of origin and source of isolation, differ from other strains by the acquisition of the GI19606 genomic island, Tn*6551* and the vB_AbaS_LC1 prophage (Figure 6), consistent with their phylogenetic placement in the same branch. All of the Japanese strains contain a copy of the small plasmid pRAY* (Figure 6 and Table 5). Variants of the small plasmid pRAY carry the *aadB* kanamycin, gentamicin and tobramycin resistance genes. So far, the pRAY* variant has been mainly associated with members of global clone 1 [13,31]. The presence of pRAY* in all Japanese strains here (Figure 6 and Table 5) suggests a local acquisition, as the other strains in this subclade, which are from different countries, do not contain this plasmid. The Thai strain (4300STDY7045730) in SC2 contains a copy of the tetracycline-resistant *tet39* gene. An analysis of this genome suggested that *tet39* is located in a 9-kb plasmid, which appears to have a novel genetic structure. This was not pursued further. AB 095 and AB 165, both belonging to SC2, both recovered in Pakistan, contain several antibiotic resistance genes, including *tet(B)*, *sul1*, *sul2*, *bla*_GES-11,_ *dfrA7*, *aacA4*, *oxa23* and *aphA6*, predicting resistance to tetracycline, sulfamethoxazole, extended spectrum beta-lactam, trimethoprim and tobramycin, as well as carbapenems and amikacin, respectively. Interestingly, an analysis of AB 095 and AB 165 genomes indicated that all of the resistance genes are carried on a putative conjugative plasmid that encodes the RepAci6 replication initiation protein [32,33,34]. Plasmids that encode RepAci6 represent a group of conjugative plasmids that are mainly associated with the spread of the *aphA6* amikacin and *oxa23* carbapenem resistance genes [32,33,34]. They often carry *aphA6* in a transposon called Tn*aphA6* and *oxa23* either in Tn*2006* or Tn*2006* embedded in the AbaR4 resistance islands [32,33,34]. Our analysis predicted a copy of Tn*aphA6* and Tn*2006* on a RepAci6 plasmid in AB 095 and AB 165. Tn*aphA6* was found precisely in the location previously identified in pAb-G7-2 (GenBank acc. no. KF669606) [33], and Tn*2006* was in the location as in pK50 (GenBank acc. no. LT984690). The remainder resistance genes—*tet(B)*, *sul1*, *sul2*, *bla*_GES-11,_ *dfrA7* and *aacA4*—were found in a MITE (Miniature Inverted-repeat Transposable Elements) region similar to that previously reported in p1AB5075 (GenBank acc. no. CP008707.1), which also encodes RepAci6 [35]. Together, the analysis of the antibiotic resistance genes in ST52 strains indicates that recent isolates have all become resistant to multiple antibiotics via different plasmids, further highlighting the significance of these mobile elements in the acquisition and spread of antibiotic resistance genes. The capsule and outer core surface polysaccharides (K and OC) loci are amongst the most variable genomic regions of *A. baumannii* genomes, even in closely related strains [30,36]. While the majority of ST52 strains encode the KL3 and OCL1 variants, WE2714, representing SC1, was found to contain KL32 and OCL6. A single strain in SC2 was also differed from other strains by a KL3àKL57 replacement (Figure 6).

## 4. Discussion

*A. baumannii* has emerged as an important opportunistic pathogen frequently associated with nosocomial infections—in particular, in intensive care units and in immunocompromised patients [1,2]. *A. baumannii* ATCC 19606 has been extensively used as a reference or model organism in studies involving the antibiotic resistance, virulence and pathogenesis of *A. baumannii* [37,38,39,40,41]. It is one of the earliest (isolated <1948) strains [5] available in current collections, making it an important strain for studies that involve antibiotic resistance. Access to high-quality complete genomes is especially important, as it provides insight into the malleability of plastic genomes; yet, until recently a complete genome for ATCC 19606 was not available. Thus far, there has also been no reported evidence of the evolutionary relationships and properties of members of ST52, a rare sequence type to which ATCC 19606 belongs. Such evidence can illuminate mechanisms for the development of antibiotic resistance, gene transfer between bacteria and increase of the resolution of outbreak tracing. Here, we report the characterisation of the first ATCC 19606 complete genome to be deposited in GenBank (October 2019) and present an analysis of its phenotypic and genomic features, as well as its evolutionary relationships to other ST52 strains.

In addition to the chromosome, our complete genome contains two cryptic plasmids, one of which (p2ATCC19606 or pMAC) was characterised in 2006 [27]. The ATCC 19606 genome completed by Zhu et al. [9] was missing the small 7-kbp cryptic plasmid (here, called p1ATCC19606), while the size and sequence of the second plasmid p2ATCC19606 (pMAC) was identical to our p2ATCC19606 sequence, as well as the original pMAC sequence (GenBank acc. no. AY541809.1). Although the ATCC 19606 genome completed by Tsubouchi et al. [10] contained p1ATCC19606 and p2ATCC19606, our analysis indicated both plasmids are likely to contain sequencing/assembling errors. In addition, given a large number of SNDs and short in/dels in this genome (GenBank acc. no. AP022836), and that p1ATCC19606 is missing from the genome completed by Zhu et al. [9], it shows that our assembly reported here is the most accurate genome for future studies of this strain.

In *A. baumannii*, antibiotic resistance genes are predominantly, although not exclusively, found within a large chromosomal genomic island. However, in more recent *A. baumannii* isolates, plasmids are significant in carrying and spreading antibiotic resistance genes [42,43,44,45]. We recently analysed a set of closely related carbapenem-resistant isolates that belong to GC1, lineage 2 and showed that they have become resistant to several antibiotics via six plasmids [46]. Similarly, here, we showed that more recent ST52 strains have also acquired genes conferring a resistance to several antibiotics via several plasmids, yet again highlighting the significance of these mobile elements in the acquisition and spread of antibiotic resistance determinants.

We characterised a novel cadmium/zinc/mercury transposon, Tn*6551,* in ATCC 19606 and showed it is related to Tn*6018*, a transposon commonly found in AbaR-type resistance islands in members of global clone 1 [3]. We previously showed that GC1 strains can gain ISAba125- and ISAba1-activated *ampC* genes [23] or an entire genomic island [22], along with a surrounding segment of the chromosome, by horizontal transfer via homologous recombination from an exogenous source. Here, we report another example where Tn*6551*, along with its flanking sequences (approx. 17 kbp in total), were exchanged between ATCC 19606 and a ST638 strain, AB301.

To treat multi-resistant infections caused by *A. baumannii* strains, many studies have begun to characterise nonantibiotic approaches, including phage therapy, which has led to the characterisation of several phages with the potential to treat *A. baumannii* infections [47]. Here, we characterised a novel prophage, named vB_AbaS_LC1, which belongs to the Siphoviridae family, in ATCC 19606, with a potential to be used in phage therapy, although further work is required to confirm this.

Here, we present an accurate complete genome sequence of *A. baumannii* ATCC 19606, which can underpin future studies of *A. baumannii*. We also showed that ST52, to which ATCC 19606 belongs, is rare, with only 13 representatives in the GenBank nonredundant and WGS databases. Despite the popularity of ATCC 19606 as a model—driven initially by the early isolation and antibiotic susceptibility of ATCC 19606— in studies aimed at understanding the pathogenicity and virulence of *A. baumannii,* it might now be time to move away from ubiquitous reliance on ATCC 19606 to the selective use of this strain for specific purposes, given that strains belonging to ST52 are not a common cause of infections globally.

## Figures and Tables

**Table 1:** Antibiotic resistance profile of ATCC 19606.

Antibiotic Family	Antibiotic	Growth Inhibition Zone (Diameter in mm) ^1^
ß-lactams	**Ampicillin**	**0**
	Cefotaxime	15
	Ceftazidime	18
	Ceftriaxone	17
	Imipenem	19
	Meropenem	21
	Ampicillin/Sulbactam	19
Aminoglycosides	Gentamicin	14
	Kanamycin	15
	Neomycin	14
	Tobramycin	13
	Netilmicin	16
	Amikacin	15
	**Streptomycin**	**7**
	**Spectinomycin**	**0**
Fluoroquinolones	Nalidixic acid	13
	Ciprofloxacin	16
Tetracyclines	Tetracycline	15
Chloramphenicol	**Chloramphenicol**	**0**
RNA Synthesis inhibitor	Rifampicin	14
Folic acid Synthesis Inhibitors	Trimethoprim	12
	**Sulfamethoxazole**	**0**

^1^ Bold indicate antibiotics to which the resistance was observed.

**Table 2:** Properties of the complete ATCC 19606 genomes.

ATCC 19606 Genomes	Length	GC%	Reading Frames	Sequencing Technology	Assembly Program	GenBank no.	Ref.
Chromosome	3,981,941	39.1	3727	Illumina MiSeq & MinION	Unicycler	CP045110	This study
p1ATCC 19606	7655	33.3	14			CP045108	
p2ATCC19606 (pMAC)	9540	34.6	14			CP045109	
Chromosome	3,980,848	39.1	3709	PacBio	SMRT	CP046654	[9]
pMAC	9540	34.6	14		plasmidSPAdes	CP046655	
Chromosome	3,978,812			Illumina MiSeq & MinION	Unicycler	AP022836	[10]
pATCC 19606-1	9408	34.6	14			AP022837	
pATCC 19606-2	7631	33.3	12			AP022838	

**Table 3:** Distribution of Tn*6551* and related transposons in *Acinetobacter*.

Genus/Species	Strain	Date	Country	Source	ST (IP)	Tn	TSD 5′-3′ ^1^	GenBank no.
*A. baumannii*	ATCC 19606	<1948	USA	Urine	52	*6551*	ATTTTTTT	CP045110
*A. baumannii*	ab736	2015	USA	Blood	52	*6551*	ATTTTTTT	CP015121
*A. baumannii*	AB031	2010	Canada	Blood	638	*6551*	ATTTTTTT	CP009256
*A. pittii*	BEC1-S18-ESBL-01	2018	Japan	Water ^2^	457	*6551*	ATTTTTTT	AP022302
*A. baumannii*	NCTC7364	2014	UK	nk ^3^	494	*6551-v1* ^4^	ATTTTTTT	LT605059
*A. indicus*	Yy_1	2019	China	Soil	-	*6551-v1*	ATTTTTTT	CP039031
*A. junii*	WCHAJ59	2015	China	Sewage	-	*6551-v1*	ATTTTTTT	CP028800
*A. nosocomialis*	AC1530	2015	Malaysia	Blood	-	*6551-v1*	ATAATTAA	CP045560
*Neisseria brasiliensis*	N.177.16	2016	Brazil	Ulcer exudate	-	*6551-v1*	ATGTATTT	CP046027

^1^ TSD, target site duplication. ^2^ Oceanic water. ^3^ Not known. ^4^ Includes a 96-bp-longer *mer* module.

**Table 4:** Annotation and function of the 59 proteins identified in the phage vB_AbaS_LC1.

Predicted Function	Biological Function	Protein Id ^1^	E-Value	Tool
Hypothetical protein	Unknown function	QFQ04715.1	4.00 × 10^−177^	BLASTp
Hypothetical protein	Unknown function	QFQ04716.1	5.00 × 10^−114^	BLASTp
Hypothetical protein	Unknown function	QFQ04717.1	4.00 × 10^−53^	BLASTp
Hypothetical protein	Unknown function	QFQ04718.1	0	BLASTp
Hypothetical protein	Unknown function	QFQ04719.1	1.00 × 10^−42^	BLASTp
RecA protein; Recombination, Radio-resistance, DNA repair, ATPase, DNA binding; HET: AGS; 2.5A {*Deinococcus radiodurans*} SCOP: d.48.1.1, c.37.1.11	Recombination	QFQ04720.1	1.30 × 10^−10^	HHpred
Hypothetical protein	Unknown function	QFQ04721.1	1.00 × 10^−73^	BLASTp
50S ribosomal protein L2, 50S; Ribosome, bacterial ribosome, proline-rich antimicrobial;	Protein synthesis	QFQ04722.1	0.3	HHpred
Hypothetical protein	Unknown function	QFQ04723.1	1.00 × 10^−102^	BLASTp
Helix-turn-helix domain-containing protein	Lysogeny	QFQ04724.1	0	BLASTp
Cro/Cl family transcriptional regulator	Lysogeny	QFQ04725.1	7.00 × 10^−54^	BLASTp
XRE family transcriptional regulator	Lysogeny	QFQ04726.1	4.00 × 10^−117^	BLASTp
Hypothetical protein	Unknown function	QFQ04727.1	7.00 × 10^−64^	BLASTp
DNA cytosine methyltransferase	Defence	QFQ04728.1	0	BLASTp
YdaU family protein	Unknown function	QFQ04729.1	0	BLASTp
Replicative DNA helicase (E.C.3.6.4.12), Bacteriophage; Helicase-loader, Helicase, DNA replication; HET: ADP; 4.1A {*Escherichia coli* O111:NM}; Related PDB entries: 6BBM_W 6BBM_V 6BBM_X 6BBM_Y	Replication	QFQ04730.1	1.20 × 10^−13^	HHpred
Hypothetical protein	Unknown function	QFQ04731.1	6.00 × 10^−80^	BLASTp
Hypothetical protein	Unknown function	QFQ04732.1	6.00 × 10^−109^	BLASTp
Hypothetical protein	Unknown function	QFQ04733.1	2.00 × 10^−75^	BLASTp
DUF1064 domain-containing protein	Unknown function	QFQ04734.1	4.00 × 10^−94^	BLASTp
Antitermination protein	Replication	QFQ04735.1	4.00 × 10^−97^	BLASTp
Hypothetical protein	Replication	QFQ04736.1	2.00 × 10^−32^	BLASTp
Hypothetical protein	Unknown function	QFQ04737.1	6.00 × 10^−164^	BLASTp
Hypothetical protein	Unknown function	QFQ04738.1	1.00 × 10^−60^	BLASTp
DNA mismatch endonuclease (E.C.3.1.-.-)/DNA complex; PROTEIN-DNA complex, mismatch, intercalation, zinc; 2.3A {*Escherichia coli*} SCOP: c.52.1.15	DNA repair	QFQ04739.1	9.50 × 10^−9^	HHpred
Hypothetical protein	Unknown function	QFQ04740.1	5.00 × 10^−159^	BLASTp
DUF2280 domain-containing protein	Unknown function	QFQ04741.1	6.00 × 10^−112^	BLASTp
Phage terminase large subunit	Replication	QFQ04742.1	0	BLASTp
DUF1073 domain-containing protein	Unknown function	QFQ04743.1	0	BLASTp
Phage head morphogenesis protein	Structural	QFQ04744.1	0	BLASTp
Possible nuclease of RNase H fold, RuvC/YqgF family	Replication	QFQ04745.1	1.00 × 10^−129^	BLASTp
Hypothetical protein	Unknown function	QFQ04746.1	5.00 × 10^−66^	BLASTp
Hypothetical protein	Unknown function	QFQ04747.1	1.00 × 10^−38^	BLASTp
Prohead core protein protease (E.C.3.4.99.-); protease pentamer, phage T4, prohead; 1.943A {Enterobacteria phage T4}; Related PDB entries: 5JBL_E 5JBL_B 5JBL_C 5JBL_A	Structural	QFQ04748.1	3.7	HHpred
Capsid fibre protein; bacteriophage, phi29, prohead, VIRUS; HET: SO4; 1.8A {Bacillus phage phi29}; Related PDB entries: 6QYY_E 6QYY_A 6QYY_D 6QYY_C 6QYY_F	Structural	QFQ04749.1	0.0013	HHpred
Capsid Stabilising Protein, Major Capsid; Major Capsid Protein, Capsid Stabilising; 3.6A {Pseudoalteromonas phage TW1}; Related PDB entries: 5WK1_A 5WK1_D 5WK1_C 5WK1_F 5WK1_E 5WK1_G	Structural	QFQ04750.1	1.30 × 10^−33^	HHpred
Mu-like prophage FluMu protein gp35; structural genomics, *Haemophilus influenzae*, hypothetical; NMR {*Haemophilus influenzae*} SCOP: l.1.1.1, d.344.1.1, a.140.3.2	Structural	QFQ04751.1	0.005	HHpred
PORTAL PROTEIN, 15 PROTEIN, HEAD; VIRAL PROTEIN, VIRAL INFECTION, TAILED; 7.2A {BACILLUS PHAGE SPP1}; Related PDB entries: 5A20_D 5A20_C 5A21_D 2KBZ_A	Structural	QFQ04752.1	0.15	HHpred
Hypothetical protein	Unknown function	QFQ04753.1	1.00 × 10^−38^	BLASTp
Hypothetical protein	Unknown function	QFQ04754.1	2.00 × 10^−111^	BLASTp
Minor tail protein U; Mixed Alpha-Beta fold, VIRAL PROTEIN; HET: MSE, SO4; 2.7A {Enterobacteria phage lambda} SCOP: d.323.1.1; Related PDB entries: 3FZ2_L 3FZ2_K 3FZ2_F 3FZB_E 3FZB_B 3FZB_A 3FZB_I 3FZB_D 3FZB_J 3FZB_H 3FZB_C 3FZ2_I 3FZB_F 3FZ2_A 3FZB_G 3FZ2_D 3FZ2_J 3FZ2_H 3FZ2_E 3FZ2_B 3FZ2_G 1Z1Z_A	Structural	QFQ04755.1	3.70 × 10^−17^	HHpred
Hypothetical protein	Unknown function	QFQ04756.1	0	BLASTp
Tail assembly chaperone; Bacteriophage HK97, morphogenesis, tail assembly; HET: MSE; 2.3A {Enterobacteria phage HK97}; Related PDB entries: 2OB9_B	Structural	QFQ04757.1	6.5	HHpred
Hypothetical protein	Unknown function	QFQ04758.1	2.00 × 10^−48^	BLASTp
Zinc ribbon domain-containing protein	Unknown function	QFQ04759.1	2.00 × 10^−117^	BLASTp
Phage tail protein	Structural	QFQ04760.1	0	BLASTp
DUF2460	Unknown function	QFQ04761.1	1.00 × 10^−161^	BLASTp
DUF2163 domain-containing protein	Unknown function	QFQ04762.1	0	BLASTp
SGNH/GDSL hydrolase family protein (sialate O-acetylesterase;cellulosome enzyme)	Lysis	QFQ04763.1	0	BLASTp
Putative Exo-alpha-sialidase; Carbohydrate-Binding Module, Bacterial Pathogen, Sialic; HET: SIA; 2.2A {CLOSTRIDIUM PERFRINGENS}; Related PDB entries: 2V73_A	Lysis	QFQ04764.1	0.0032	HHpred
C40 family peptidase	Lysis	QFQ04765.1	2.00 × 10^−98^	BLASTp
Putative phage tail protein	Structural	QFQ04766.1	0	BLASTp
Hypothetical protein	Unknown function	QFQ04767.1	4.00 × 10^−87^	BLASTp
Glycosyl hydrolase 108	Lysis	QFQ04768.1	2.00 × 10^−141^	BLASTp
Anaerobic dehydrogenase	Lysis	QFQ04769.1	7.00 × 10^−73^	BLASTp
Hypothetical protein	Unknown function	QFQ04770.1	4.00 × 10^−168^	BLASTp
Y-family DNA polymerase	Defence	QFQ04771.1	0	BLASTp
Trans-lesion error-prone DNA polymerase V autoproteolytic subunit	Defence	QFQ04772.1	6.00 × 10^−115^	BLASTp
Site-specific integrase	Lysogeny	QFQ04773.1	0	BLASTp

^1^ Protein IDs based on the complete genome of ATCC 19606, GenBank accession number CP045110.

**Table 5:** Properties of strains belonging to ST52 ^1^.

Strain	Country	Date	Isolation Source	KL	OC	GI19606	Additional Resistance Genes ^2^	Tn*6551*	Φ ^3^	p1/p2 ^4^	GenBank no.
ATCC 19606	US	<1948	Urine	3	1	Y	*−*	+	+	+/+	CP045110
MSP4-16	India	2010	Mangrove soil	3	1	Y	*−*	+	+	+/+	AODW01
ab736	US	2015	Blood	3	1	Y	*−*	+	+	−/+	CP015121
NBRC 110495	Japan	2008	Human abscess	3	1	−	*aadB* ^5^	−	−	−/+ ^6^	BBOR01
NBRC 110494	Japan	2008	Burned skin	3	1	−	*aadB* ^5^	−	−	−/+ ^6^	BBTE01
NBRC 110493	Japan	2008	Burned skin	3	1	−	*aadB* ^5^	−	−	−/+ ^6^	BBOQ01
GTC 03329	Japan	2008	Human abscess	3	1	−	*aadB* ^5^	−	−	−/+ ^6^	BBNJ01
GTC 03325	Japan	2008	Burned skin	3	1	−	*aadB* ^5^	−	−	−/+ ^6^	BBSP01
GTC 03324	Japan	2008	Burned skin	3	1	−	*aadB* ^5^	−	−	−/+ ^6^	BBNH01
AB_095	Pakistan	2016	ICU washroom sink	3	1	−	*tet(B), sul1, sul2, bla*_GES-11,_ *dfrA7, aacA4, oxa23, aphA6*	−	−	−/−	RHZR01
AB_165	Pakistan	2016	Alcohol foam dispenser in ICU	3	1	−	*tet(B), sul1, sul2, bla*_GES-11,_ *dfrA7, aacA4, oxa23, aphA6*	−	−	−/−	RHZA01
4300STDY7045730	Thailand	2016	na ^7^	57	1	−	*tet39*	−	−	−/−	UFJF01
WE2714	China	na	na	32	6	−	*−*	−	−	−/−	QKVH01

^1^ All strains encode the OXA-694 variant of the intrinsic *oxa-Ab* gene. ^2^ Additional antibiotic resistance genes (in addition to sul2, which is in the GI*sul2* genomic island). ^3^ vB_AbaS_LC1. ^4^ p1, p1ATCC 19606 and p2, p2ATCC19606 (pMAC). ^5^ *aadB* in plasmid pRAY*. ^6^ An approx. 6-kb variant of p2ATCC 19606 (pMAC) present. ^7^ Not available.

## Supplementary Materials

The following are available online at https://www.mdpi.com/2076-2607/8/12/1851/s1: Table S1. Bacterial strains employed to determine the host range of the phage vB_AbaS_LC1.

## References

[1] Peleg A.Y., Seifert H., Paterson D.L. (2008). *Acinetobacter baumannii*: Emergence of a successful pathogen. Clin. Microbiol. Rev..

[2] Harding C.M., Hennon S.W., Feldman M.F. (2018). Uncovering the mechanisms of *Acinetobacter baumannii* virulence. Nat. Rev. Microbiol..

[3] Hamidian M., Hall R.M. (2018). The AbaR antibiotic resistance islands found in *Acinetobacter baumannii* global clone 1—Structure, origin and evolution. Drug Resist. Updates Rev. Comment. Antimicrob. Anticancer Chemother..

[4] De Gregorio E., Del Franco M., Martinucci M., Roscetto E., Zarrilli R., Di Nocera P.P. (2015). Biofilm-associated proteins: News from *Acinetobacter*. BMC Genom..

[5] Hugh R., Reese R. (1968). A comparison of 120 strains of *Bacterium anitratum* Schaub and Hauber with the type strain of this species. Int. J. Syst. Evol. Microbiol..

[6] Harris G., Lee R.K., Lam C.K., Kanzaki G., Patel G.B., Xu H.H., Chen W. (2013). A mouse model of *Acinetobacter baumannii*-associated pneumonia using a clinically isolated hypervirulent strain. Antimicrob. Agents Chemother..

[7] Pachón-Ibáñez M.E., Docobo-Pérez F., López-Rojas R., Domínguez-Herrera J., Jiménez-Mejias M.E., García-Curiel A., Pichardo C., Jiménez L., Pachón J. (2010). Efficacy of rifampin and its combinations with imipenem, sulbactam, and colistin in experimental models of infection caused by imipenem-resistant *Acinetobacter baumannii*. Antimicrob. Agents Chemother..

[8] Davenport K.W., Daligault H.E., Minogue T.D., Bruce D.C., Chain P.S., Coyne S.R., Jaissle J.G., Koroleva G.I., Ladner J.T., Li P.E. (2014). Draft Genome Assembly of *Acinetobacter baumannii* ATCC 19606. Genome Announc..

[9] Zhu Y., Lu J., Zhao J., Zhang X., Yu H.H., Velkov T., Li J. (2020). Complete genome sequence and genome-scale metabolic modelling of *Acinetobacter baumannii* type strain ATCC 19606. Int. J. Med. Microbiol..

[10] Tsubouchi T., Suzuki M., Niki M., Oinuma K.I., Niki M., Kakeya H., Kaneko Y. (2020). Complete Genome Sequence of *Acinetobacter baumannii* ATCC 19606(T), a Model Strain of Pathogenic Bacteria Causing Nosocomial Infection. Microbiol. Resour. Announc..

[11] Wiegand I., Hilpert K., Hancock R.E. (2008). Agar and broth dilution methods to determine the minimal inhibitory concentration (MIC) of antimicrobial substances. Nat. Protoc..

[12] CLSI (2012). Performance Standards for Antimicrobial Susceptibility Testing.

[13] Hamidian M., Hall R.M. (2018). Genetic structure of four plasmids found in *Acinetobacter baumannii* isolate D36 belonging to lineage 2 of global clone 1. PLoS ONE.

[14] O’Toole G.A. (2011). Microtiter dish biofilm formation assay. J. Vis. Exp..

[15] Gaio D., To J., Liu M., Monahan L., Anantanawat K., Darling A.E. (2019). Hackflex: Low cost Illumina sequencing library construction for high sample counts. bioRxiv.

[16] Wick R.R., Judd L.M., Gorrie C.L., Holt K.E. (2017). Completing bacterial genome assemblies with multiplex MinION sequencing. Microb. Genom..

[17] Wick R.R., Judd L.M., Gorrie C.L., Holt K.E. (2017). Unicycler: Resolving bacterial genome assemblies from short and long sequencing reads. PLoS Comput. Biol..

[18] Seemann T. (2014). Prokka: Rapid prokaryotic genome annotation. Bioinformatics.

[19] Carver T.J., Rutherford K.M., Berriman M., Rajandream M.A., Barrell B.G., Parkhill J. (2005). ACT: The Artemis Comparison Tool. Bioinformatics.

[20] Kropinski A.M., Mazzocco A., Waddell T.E., Lingohr E., Johnson R.P. (2009). Enumeration of bacteriophages by double agar overlay plaque assay. Methods Mol. Biol..

[21] Hamidian M., Hall R.M. (2017). *Acinetobacter baumannii* ATCC 19606 Carries GI*sul2* in a Genomic Island Located in the Chromosome. Antimicrob. Agents Chemother..

[22] Hamidian M., Hawkey J., Wick R., Holt K.E., Hall R.M. (2019). Evolution of a clade of *Acinetobacter baumannii* global clone 1, lineage 1 via acquisition of carbapenem- and aminoglycoside-resistance genes and dispersion of ISAba1. Microb. Genom..

[23] Hamidian M., Hall R.M. (2014). Resistance to third-generation cephalosporins in *Acinetobacter baumannii* due to horizontal transfer of a chromosomal segment containing ISAba1-*ampC*. J. Antimicrob. Chemother..

[24] Blackwell G.A., Hall R.M. (2017). The *tet39* Determinant and the *msrE-mphE* Genes in *Acinetobacter* Plasmids Are Each Part of Discrete Modules Flanked by Inversely Oriented pdif (XerC-XerD) Sites. Antimicrob. Agents Chemother..

[25] D’Andrea M.M., Giani T., D’Arezzo S., Capone A., Petrosillo N., Visca P., Luzzaro F., Rossolini G.M. (2009). Characterization of pABVA01, a plasmid encoding the OXA-24 carbapenemase from Italian isolates of *Acinetobacter baumannii*. Antimicrob. Agents Chemother..

[26] Merino M., Acosta J., Poza M., Sanz F., Beceiro A., Chaves F., Bou G. (2010). OXA-24 carbapenemase gene flanked by XerC/XerD-like recombination sites in different plasmids from different Acinetobacter species isolated during a nosocomial outbreak. Antimicrob. Agents Chemother..

[27] Dorsey C.W., Tomaras A.P., Actis L.A. (2006). Sequence and organization of pMAC, an *Acinetobacter baumannii* plasmid harboring genes involved in organic peroxide resistance. Plasmid.

[28] Tomaras A.P., Dorsey C.W., Edelmann R.E., Actis L.A. (2003). Attachment to and biofilm formation on abiotic surfaces by *Acinetobacter baumannii*: Involvement of a novel chaperone-usher pili assembly system. Microbiology.

[29] Wyres K.L., Cahill S.M., Holt K.E., Hall R.M., Kenyon J.J. (2020). Identification of *Acinetobacter baumannii* loci for capsular polysaccharide (KL) and lipooligosaccharide outer core (OCL) synthesis in genome assemblies using curated reference databases compatible with Kaptive. Microb. Genom..

[30] Kenyon J.J., Hall R.M. (2013). Variation in the complex carbohydrate biosynthesis loci of *Acinetobacter baumannii* genomes. PLoS ONE.

[31] Hamidian M., Nigro S.J., Hall R.M. (2012). Variants of the gentamicin and tobramycin resistance plasmid pRAY are widely distributed in *Acinetobacter*. J. Antimicrob. Chemother..

[32] Hamidian M., Hall R.M. (2014). pACICU2 is a conjugative plasmid of *Acinetobacter* carrying the aminoglycoside resistance transposon Tn*aphA6*. J. Antimicrob. Chemother..

[33] Hamidian M., Holt K.E., Pickard D., Dougan G., Hall R.M. (2014). A GC1 *Acinetobacter baumannii* isolate carrying AbaR3 and the aminoglycoside resistance transposon Tn*aphA6* in a conjugative plasmid. J. Antimicrob. Chemother..

[34] Hamidian M., Kenyon J.J., Holt K.E., Pickard D., Hall R.M. (2014). A conjugative plasmid carrying the carbapenem resistance gene *bla*OXA-23 in AbaR4 in an extensively resistant GC1 *Acinetobacter baumannii* isolate. J. Antimicrob. Chemother..

[35] Gallagher L.A., Ramage E., Weiss E.J., Radey M., Hayden H.S., Held K.G., Huse H.K., Zurawski D.V., Brittnacher M.J., Manoil C. (2015). Resources for Genetic and Genomic Analysis of Emerging Pathogen *Acinetobacter baumannii*. J. Bacteriol..

[36] Holt K., Kenyon J.J., Hamidian M., Schultz M.B., Pickard D.J., Dougan G., Hall R. (2016). Five decades of genome evolution in the globally distributed, extensively antibiotic-resistant *Acinetobacter baumannii* global clone 1. Microb. Genom..

[37] Breisch J., Waclawska I., Averhoff B. (2019). Identification and characterization of a carnitine transporter in *Acinetobacter baumannii*. MicrobiologyOpen.

[38] Moffatt J.H., Harper M., Harrison P., Hale J.D., Vinogradov E., Seemann T., Henry R., Crane B., St Michael F., Cox A.D. (2010). Colistin resistance in *Acinetobacter baumannii* is mediated by complete loss of lipopolysaccharide production. Antimicrob. Agents Chemother..

[39] Sato Y., Unno Y., Kawakami S., Ubagai T., Ono Y. (2017). Virulence characteristics of *Acinetobacter baumannii* clinical isolates vary with the expression levels of omps. J. Med. Microbiol.

[40] Zeidler S., Müller V. (2019). The role of compatible solutes in desiccation resistance of *Acinetobacter baumannii*. MicrobiologyOpen.

[41] Zimbler D.L., Arivett B.A., Beckett A.C., Menke S.M., Actis L.A. (2013). Functional features of TonB energy transduction systems of *Acinetobacter baumannii*. Infect. Immun..

[42] Blackwell G.A., Hall R.M. (2019). Mobilisation of a small *Acinetobacter* plasmid carrying an *oriT* transfer origin by conjugative RepAci6 plasmids. Plasmid.

[43] Hamidian M., Ambrose S.J., Hall R.M. (2016). A large conjugative *Acinetobacter* baumannii plasmid carrying the *sul2* sulphonamide and *strAB* streptomycin resistance genes. Plasmid.

[44] Leungtongkam U., Thummeepak R., Tasanapak K., Sitthisak S. (2018). Acquisition and transfer of antibiotic resistance genes in association with conjugative plasmid or class 1 integrons of *Acinetobacter baumannii*. PLoS ONE.

[45] Ma F., Shen C., Zheng X., Liu Y., Chen H., Zhong L., Liang Y., Liao K., Xia Y., Tian G.B. (2019). Identification of a Novel Plasmid Carrying *mcr-4.3* in an *Acinetobacter baumannii* Strain in China. Antimicrob. Agents Chemother..

[46] Douraghi M., Kenyon J.J., Aris P., Asadian M., Ghourchian S., Hamidian M. (2020). Accumulation of Antibiotic Resistance Genes in Carbapenem-Resistant *Acinetobacter baumannii* Isolates Belonging to Lineage 2, Global Clone 1, from Outbreaks in 2012-2013 at a Tehran Burns Hospital. mSphere.

[47] García-Quintanilla M., Pulido M.R., López-Rojas R., Pachón J., McConnell M.J. (2013). Emerging therapies for multidrug resistant *Acinetobacter baumannii*. Trends Microbiol..

