# Supplementary material for: Analysis of Complete Genome Sequence of Acinetobacter baumannii Strain ATCC 19606 Reveals Novel Mobile Genetic Elements and Novel Prophage
Source: Microorganisms. 2020 Nov 24;8(12):1851. doi: 10.3390/microorganisms8121851 (PMC7760358; doi:10.3390/microorganisms8121851)
Supplement: Supplementary file 1 [file microorganisms-08-01851-s001.pdf]

Table S1. Bacterial strains employed to determine the host range of the phage vB_AbaS_LC1.

Strains	ST[1]	Spot [2]
Ab05_GEIH2010	186	-
Ab09_GEIH2010	297	-
Ab105_GEIH2010	2	-
Ab13_GEIH2010	79	-
Ab155_GEIH2000	2	-
Ab177_GEIH2000	2	-
Ab217_GEIH2010	2	-
Ab22_GEIH2010	52	-
Ab226_GEIH2010	181	-
Ab24_GEIH2010	255	+
Ab291_GEIH2010	265	+
Ab309_GEIH2010	267	-
Ab310_GEIH2010	268	-
Ab32_GEIH2010	2	-
Ab404_GEIH2010	80	-
Ab421_GEIH2010	2	-
Ab440_GEIH2010	187	-
Ab461_GEIH2010	2	-
Ab464_GEIH2010	272	-

[1] ST: Sequence Type.
[2] Spot Test: clear spot (+), non spot (-).
